# Embryological evidence substantiates the subcoxal theory on the origin of pleuron in insects

**DOI:** 10.1038/s41598-017-12728-2

**Published:** 2017-10-03

**Authors:** Yuta Mashimo, Ryuichiro Machida

**Affiliations:** 1 Sugadaira Research Station, Mountain Science Center, University of Tsukuba, Sugadaira Kogen 1278-294, Ueda, Nagano, 386-2204 Japan; 2 Present Address: Graduate School of Symbiotic Systems Science and Technology, Fukushima University, Kanayagawa 1, Fukushima, Fukushima, 960-1296 Japan

## Abstract

The lateral body plate pleuron is a significant structure in insects that contributes to the development and elaboration of wings and limbs (appendages). Although the pleuron is thought to originate from the proximal-most appendicular segment, the subcoxa, details remain unclear, and the morphological boundary between the dorsal body plate tergum and appendage (BTA) has not been clearly specified. Employing low-vacuum scanning electron microscopy (SEM) and the nano-suit method for SEM, we followed, in detail, the development of the thoracic segments of the two-spotted cricket *Gryllus bimaculatus* and succeeded in clearly defining the BTA. This study demonstrates the subcoxal origin of the pleuron, suggests the tergal origin of spiracles, and reveals that the wing proper originates exclusively from the tergum, whereas the wing hinge and direct muscles may be appendicular in origin, suggesting the dual origin (i.e., tergal plus appendicular origin) of wings.

## Introduction

Insects are the most successful group of animals, with one million known species at present, and their megadiversity has attracted researchers’ attention. The striking success of insects is due to multiple factors. For example, differentiation of the life cycle, e.g., holometaboly, leads to the establishment of enormous niches. Insects also have exoskeletons that function as a barrier to water loss, enabling the exploration of terrestrial habitat and providing a tough attachment point for strong musculature. Their tripartite body structure, composed of a cephalic tagma specialized for feeding and integration, a thoracic component for locomotion, and an abdominal component for digestion and reproduction result in incredibly high performance^1–3^. Focusing on the thorax, which is composed of three segments, insects have diverse limb types that function in various ways for each group. Pterygota, or insects that have developed wings, account for 99% of insects. Insects with wings are able to move rapidly within and between habitats. Thoracic segments include three exoskeletal elements: the dorsal tergum, the lateral pleuron, and the ventral sternum. Thoracic limbs have elastic bases in joints on the pleuron, which also provide mechanical support to limb movement and provide a wide variety of functions for insects. Wings, which provide insects with new aerial habitats, are elastically hinged on the pleuron on the ventral side; this pleuron is robust enough to ensure attachment of strong flight muscles. Therefore, as Coulcher *et al*.^4^ mentioned, the pleuron plays an important role in providing mechanical support for limbs and wings, driven by highly developed muscles, as well as in providing elastic joints or hinges against the trunk.

In spite of its importance, the pleuron has not been well investigated or understood. The pleuron is composed of two major parts, the anterior episternum and posterior epimeron. Heymons^5^ suggested, in his classical embryological work on the aquatic hemipteran *Naucoris* sp., that the pleuron originates from the proximal podomere (limb segment), the subcoxa. This concept has been refined as the “subcoxal theory on the origin of pleuron” (hereafter, the subcoxal theory) by morphologists, such as Snodgrass^6,7^, Weber^8^, and Matsuda^9^. A recent molecular developmental study by Coulcher *et al*.^4^ is noteworthy. They distinguished five domains of the gene *serrate* homolog (*Tc-ser*) expression in the embryonic appendages (limbs) of the red flour beetle *Tribolium castaneum*, which correspond to five joints separating six segments, i.e., subcoxa, coxa, trochanter, femur, tibiotarsus, and pretarsus. This is the first molecular evidence for the existence of subcoxa. They also suggested that the subcoxa is involved in the formation of the pleuron and advocated the subcoxal theory. Finally, satisfactory evidence seems have been presented for the subcoxal theory.

**Figure 1:**
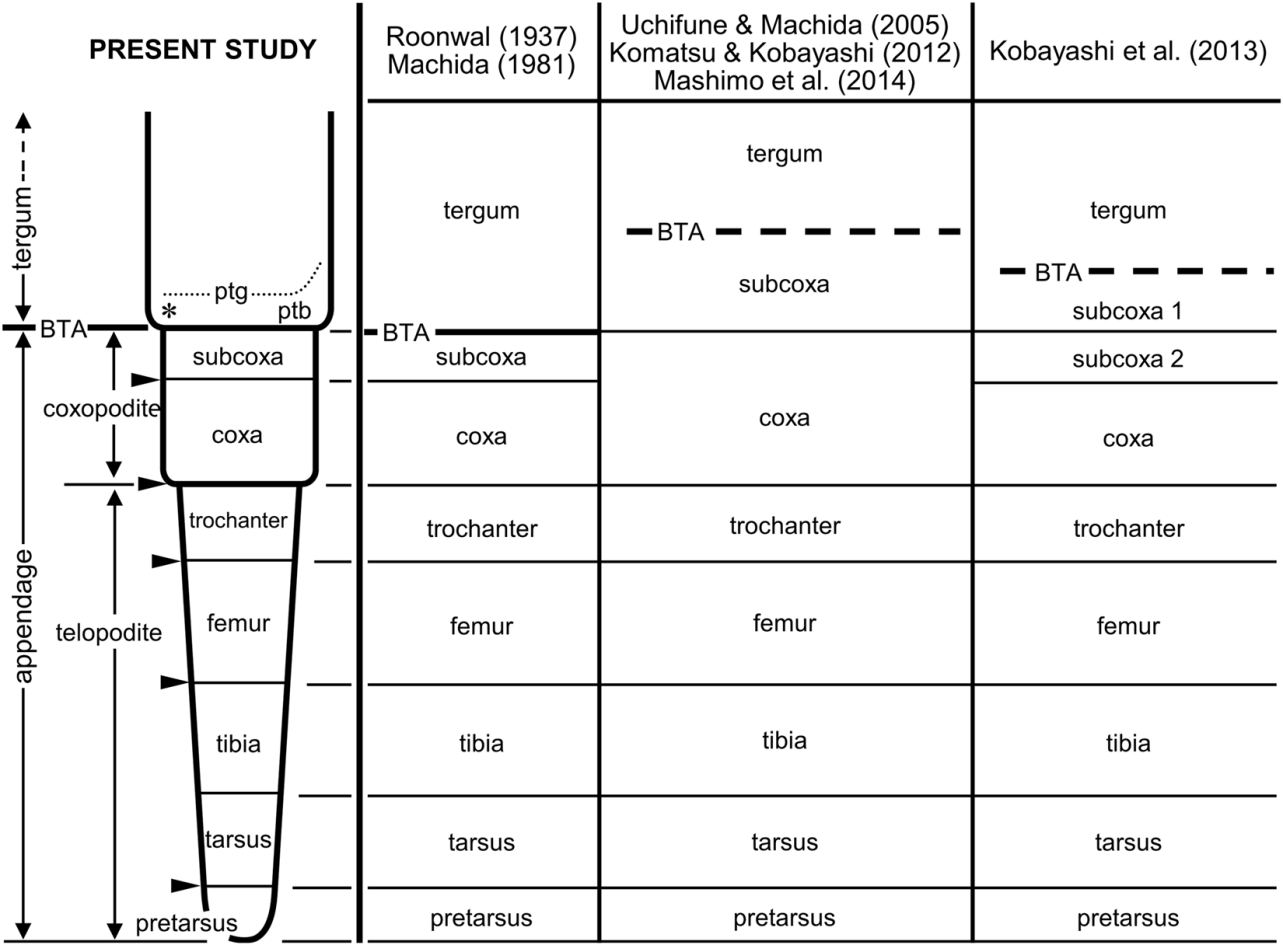
Segmentation of appendage with special reference to identification of the proximal podomeres and the boundary between the tergum and the appendage: conclusions from this embryological study on *Gryllus bimaculatus* and comparisons with previous studies. Abbreviations: BTA, boundary between tergum and appendage; ptb, paratergal bulge; ptg, paratergal groove. Asterisk and arrowheads show the spiracle and domains of *Tc-ser* expression in Coulcher *et al*.^4^, respectively.

However, morphological evidence for the subcoxal theory remains unclear. The definition of the boundary between the embryonic subcoxa and tergum, or their extension, varies between researchers (Fig. 1). Despite this, researchers support the subcoxal theory^10–13^. To date, it is unclear how much the subcoxa contributes to the formation of the pleuron. For example, spiracles are located in the epipleural field or the dorsal pleuritis, which are fragmented sclerites of pleuron. Are spiracles derived from the subcoxa or are they tergal in origin? Such confusion may be due to the lack of reliable embryological evidence, as previously described by Matsuda^9^ “*It is clear … that workers, including the present writer, have tended to indulge in speculations, extending and modifying the subcoxal theory, without really looking for reliable developmental facts in support*.” Recent embryological studies, including Uchifune and Machida^11^ using a grylloblattodean and Kobayashi *et al*.^12^ using an adephagan beetle, tried to strictly re-evaluate the subcoxal theory with scanning electron microscopy (SEM). However, they failed to follow the detailed transformation of embryonic subcoxa into the definitive pleuron, as in previous embryological studies by Roonwal^10^, Ibrahim^14^, and Bretfeld^15^. The reason for this failure may be very simple. Although SEM provides high-resolution images, the cuticle secreted during the late embryonic stage is a significant obstacle to observing embryonic surface structure. Due to the processing necessary for standard high vacuum SEM imaging, fine embryonic cuticles are often swollen and separated from the embryo proper; this disables the capturing of the true image of embryo. The thin and elastic cuticle of first instar nymphs also creates problems in observation. The processing of materials, such as fixation, dehydration, and drying, shrinks specimens, distorts the nymphal cuticle and makes an accurate understanding of the surface structure impossible.

To surmount these problems, we used low-vacuum SEM and the nano-suit method for SEM. As Machida^16^ suggested, the former enables us to observe the true surface image of non-coated embryos through the wrinkled embryonic cuticle. The latter makes it possible for us to observe artifact-free specimens using SEM, such as non-fixed living specimens and non-dried specimens^17,18^. Utilizing these two methods, we followed the detailed formation of the pleuron throughout development in the two-spotted cricket *Gryllus bimaculatus* to provide a sound basis for discussions on the subcoxal theory and related issues.

## Results and Discussion

Staging for the embryonic development of *G. bimaculatus* was performed as described by Niwa *et al*.^19^.

### Morphogenesis of the thorax in *Gryllus bimaculatu*s

**Figure 2:**
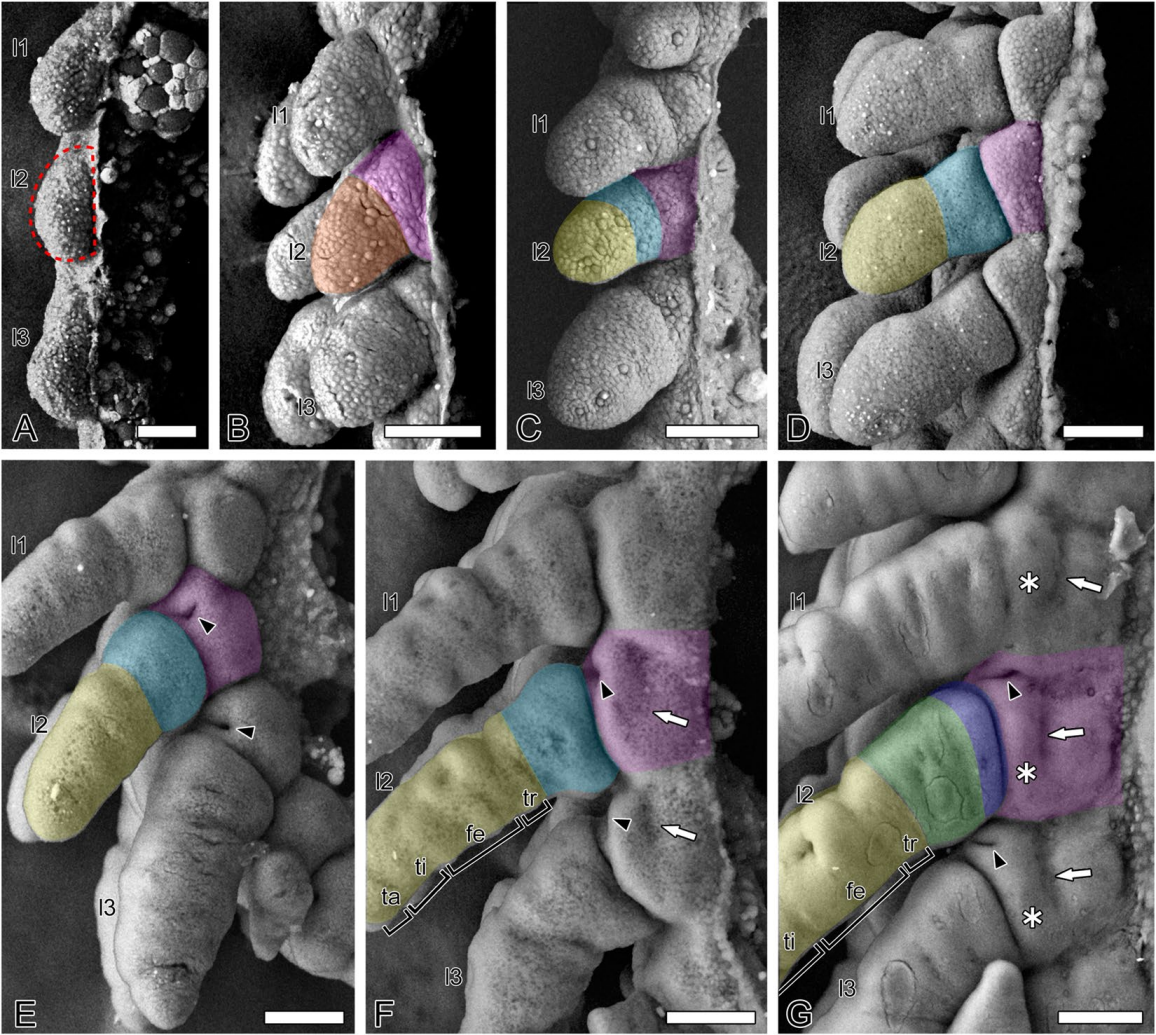
Low-vacuum SEMs of *Gryllus bimaculatus* embryos, lateral views. (**A**) Stage 5. Dashed line indicates the lateral bulge of the segment. (**B**) Stage 6. (**C**) Early Stage 7. (**D**) Late Stage 7. (**E**) Stage 8. (**F**) Early Stage 9. (**G**) Late Stage 9. Abbreviations: fe, femur; l1-3, pro-, meso- and metalimbs; ta, tarsus; ti, tibia; tr, trochanter. Color highlights: magenta, tergum; orange, appendage; light blue, coxopodite; dark blue, subcoxa; green, coxa; yellow, telopodite. Black arrowheads, white arrows, and asterisks show spiracles, paratergal grooves, and bulges, respectively. Bars: 50 µm.

The process of segmentation and the bulging of lateral areas in each segment can be observed in Stage 5 (Fig. 2A). In Stage 6, the enlarging bulge is transversely demarcated into two parts (pink and orange in Fig. 2B). Recent embryological studies with SEM^11–13,20,21^ identified a demarcation identical to this as a division of two podomeres, i.e., the boundary between the coxa and subcoxa or between subcoxae 1 and 2. However, we believe this demarcation indicates differentiation of the proximal “tergum” (pink in Fig. 2B) and the distal “limb” or the “appendage” (orange in Fig. 2B), consistent with microscopy observations from Roonwal^10^ and Machida^22^ (Fig. 1). The rationale for this decision is addressed in the next section, “Subcoxal theory substantiated.” In Stage 7, thoracic appendages elongate slightly and divide into the proximal coxopodite (light blue in Fig. 2C) and the distal telopodite (yellow in Fig. 2C), and the boundary between the tergum and appendage becomes more distinct (Fig. 2D). In Stage 8, the telopodite further elongates and a spiracle, which is the invagination of trachea, arises as an ectodermal depression at the anteroventral region of each of the terga of the pterothoraxes, i.e., meso- and metathorax (black arrowheads in Fig. 2E).

In Stage 9, the telopodite subdivides into several podomeres, including the trochanter, femur, tibia, and tarsus, at the apex of which the pretarsus later differentiates (Fig. 2F). Next, the coxopodite subdivides into the proximal narrow subcoxa (dark blue in Fig. 2G) and the distal coxa (green in Fig. 2G). The tergum gradually enlarges (Fig. 2F,G). A longitudinal depression appears, called the “paratergal groove” (white arrows in Fig. 2F,G). Conversely, the region beneath the paratergal groove bulges, becoming the “paratergal bulge” (asterisks in Fig. 2G). The paratergal groove is less distinct in the prothorax than in the pterothoraxes. In previous studies on polyneopteran embryos (Grylloblattodea^11^, Embioptera^21^, and Zoraptera^13^), structures identical to the paratergal groove were identified as “pleural sutures.” However, this identification is misleading. As described later, this study reveals that the true pleural suture forms during the final developmental stage when the nymphal cuticle is secreted.

**Figure 3:**
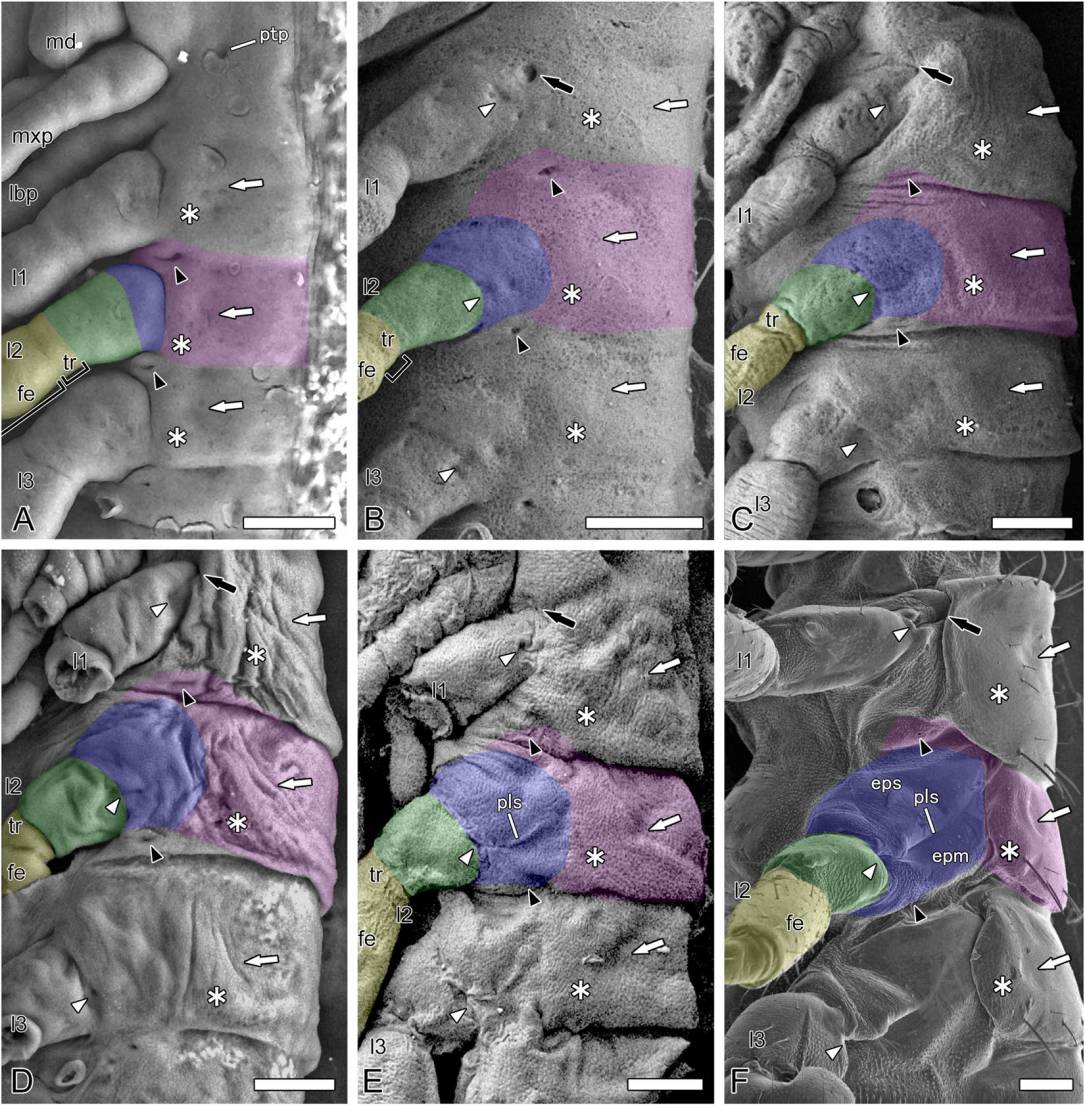
Low-vacuum SEMs of *Gryllus bimaculatus* embryos and SEM of nymph using the nano-suit method, lateral views. (**A**) Stage 10. (**B**) Stage 11. (**C**) Stage 12. (**D**) Stage 13. (**E**) Stage 14. (**F**) First instar nymph. Abbreviations: fe, femur; lbp, labial palp; l1-3, pro-, meso- and metalimbs; md, mandible; mxp, maxillary palp; pls, pleural suture; pta, posterior tentorial pit; tr, trochanter. Color highlights: dark blue, subcoxa; green, coxa; magenta, tergum; yellow, telopodite. Black arrowheads, black arrows, white arrowheads, white arrows, and asterisks show spiracles, the structure in the prothorax serially homologous to spiracles (see text), pleuro-coxal joints, paratergal grooves, and paratergal bulges, respectively. Bars: 100 µm.

**Figure 4:**
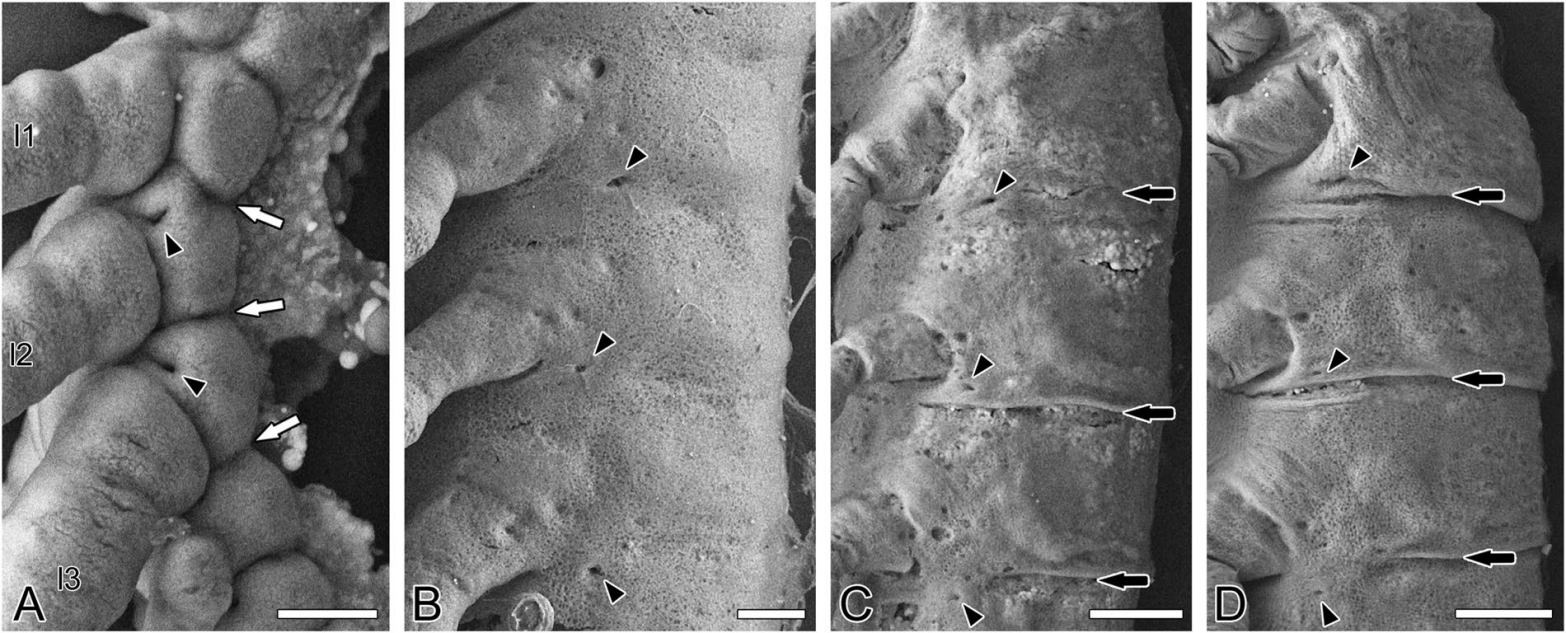
Low-vacuum SEMs of *Gryllus bimaculatus* embryos, lateral views. (**A**) Stage 8. (**B**) Stage 11. (**C**) Stage 12. (**D**) Stage 13. Black arrowheads, white arrows, and black arrows show spiracles, primary segmentation lines, and secondary ones, respectively. Bars: 50 µm (**A**,**B**) ; 100 µm (**C**,**D**).

Newly formed embryos of *G. bimaculatus* descend into the yolk during a process named anatrepsis. During the first half of development, embryos maintain a compact posture in the yolk. In Stage 10, embryos ascend to the egg’s surface through a process called katatrepsis, and are freed from the constraints of the yolk^19^. This results in rapid elongation and expansion, the surface of the embryos is temporarily stretched, and surface structures that were distinct in the previous stages, such as the paratergal groove and bulge, and structural boundaries (Figs 2G and 3A), are obliterated in Stage 11 (Figs 3B and 4B). In spite of the temporal obliteration of external features, we can follow the development from Stage 10 through Stage 12, as shown with symbols and color highlights in Fig. 3A–F.

A pit appears proximally to the subcoxa of the prothoracic limb in Stage 11 (black arrow in Fig. 3B). Formation of the pleuro-coxal joints is visible as invaginations at the subcoxo-coxal boundaries of the thoracic limbs in Stages 11–12 (white arrowheads in Fig. 3B,C). The pleuro-coxal joints are a good marker for the distal limit of the subcoxa.

**Figure 5:**
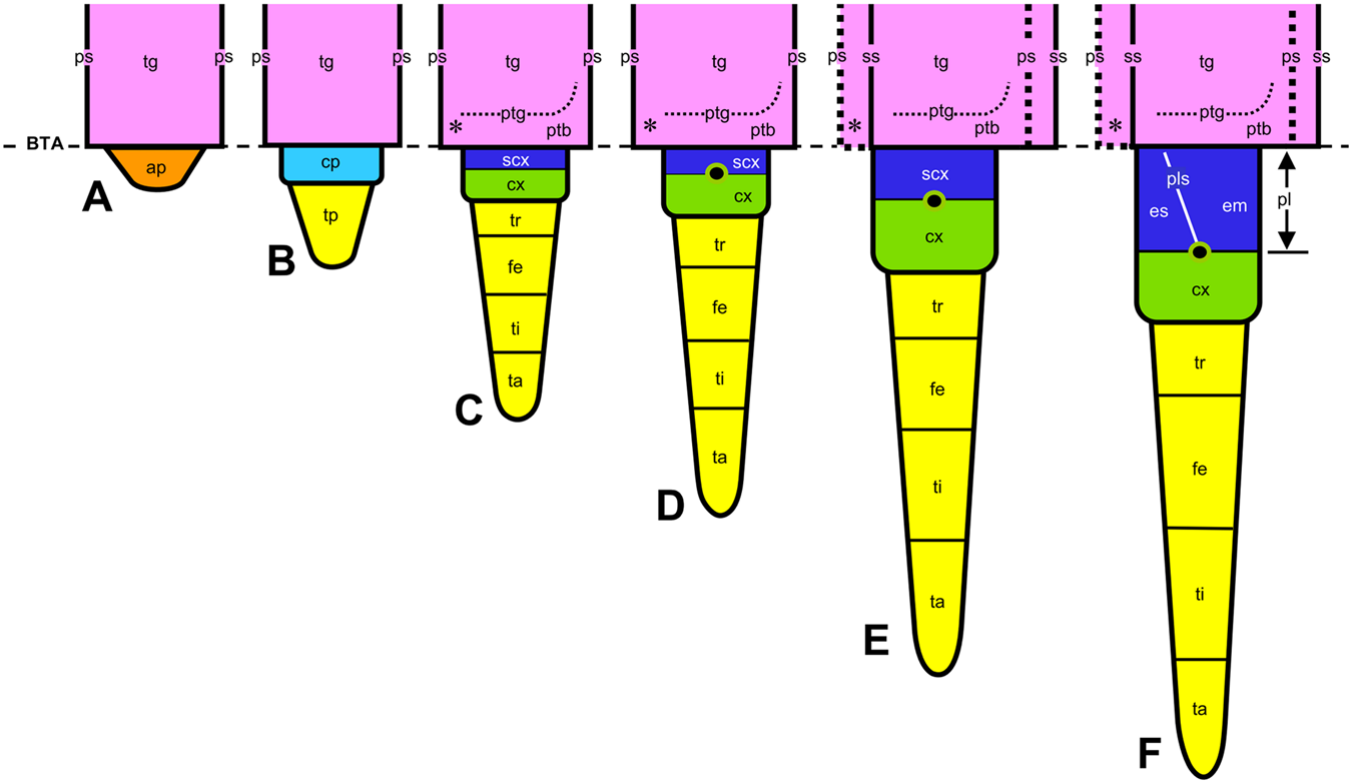
Diagrammatic summary of the morphogenesis of thoracic segments in *Gryllus bimaculatus*. (**A**) Stage 6, lateral bulge is divided by the boundary between the tergum and appendage (BTA) into the proximal tergum (tg: pink) and distal appendage (ap: orange). (**B**) Stage 7, the appendage is divided into the proximal coxopodite (cp: light blue) and distal telopodite (tp: yellow). (**C**) Stage 9, the telopodite is subdivided into the trochanter (tr), femur (fe), tibia (ti), and tarsus (ta). The coxopodite is subdivided into the proximal subcoxa (scx: dark blue) and distal coxa (cx: green). A spiracle (asterisk) appears at the anteroventral region of the tergum, and then the paratergal groove (ptg) and the paratergal bulge (ptb) appear. (**D**) Stage 11, the pleuro-coxal joint (black circle) forms in the boundary of the subcoxa and coxa. (**E**) Stage 12, the boundary of segments (boundary of the primary segmentation: ps) is obliterated, and the new segmental boundary (boundary of the primary segmentation: ss) is renewed. Pretarsus (pta) differentiates. (**F**) Stage 14, the pleural suture (pls) differentiates, which demarcates the subcoxa into the anterior episternum (es) and the posterior epimeron (em). The pleuron (pl) acquires its definitive, basic construction.

In Stage 11, just after katatrepsis is completed, the segmental boundaries are obliterated (Figs 3B and 4B). Immediately in the next Stage 12, the segmental boundaries become visible again (Figs 3C and 4C). Comparing the segmental boundaries before Stage 12 (the boundary of the primary segmentation, white arrows in Fig. 4A) and after Stage 12 (the boundary of the secondary segmentation, black arrows in Fig. 4C,D) with the spiracles (black arrowheads in Fig. 4A–D) reveals differences. The front boundary of the primary segmentation is anterior to the spiracle in the segment (Fig. 4A), whereas the boundary of the secondary segmentation is posterior to the spiracle, located in the posterior lower region of the anterior segment (Fig. 4C,D). Because of the renewal of the segmental boundaries, the spiracles have suffered an apparent topographical “shift” across the segmental boundaries (Fig. 5).

In the final developmental Stages 14 and 15, when the nymphal cuticle is secreted, the subcoxa enlarges rapidly and the “true” pleural suture differentiates, which runs obliquely through the subcoxa from the pleuro-coxal joint (Fig. 3E). In first instar nymphs, who have acquired their definitive configuration, the pleural suture clearly demarcates the subcoxal region into the anterior episternum and the posterior epimeron (Fig. 3F). The development of wings is described in the section “Dual origin of wings supported.”

### Subcoxal theory substantiated

Due to interference from the embryonic cuticle, entomologists have been unable to observe later developmental stages and the drastic morphogenesis occurring beneath the cuticle. As a result, they have provided multiple speculative perspectives on the formation of the pleuron (Fig. 1). The most problematic of these speculations may be *which demarcation in the embryo is the boundary between the tergum and the appendage or subcoxa* (BTA). Here, we followed the formation of the pleuron throughout development; from the early embryos to the first instar nymphs that represent the definitive body plan (Fig. 5) using low-vacuum SEM for embryos and the nano-suit method of SEM for first instar nymphs. We found landmarks approximately defining the lower limit of the tergum: the paratergal groove (white arrows in Figs  2F,G and 3A–F) and the paratergal bulge (asterisks in Figs 2G and 3A–F). Using these landmarks, we could identify the initial BTA in Stage 6 (between pink and orange in Fig. 2B cf. Fig. 5A), which becomes deeply incised and the most distinctive structural division in Stage 7 (between pink and light blue in Fig. 2D–F, between pink and dark blue in Fig. 2G cf. Fig. 5B,C). The subcoxa can be identified as the area shown in dark blue in Figs 2G and 3A–F, which is defined proximally by the BTA and distally by the subcoxo-coxal boundary, which is defined by the pleuro-coxal joint (white arrowheads in Fig. 3B–F cf. Fig. 5D,E). Thus, “*looking for reliable developmental facts in support”* (from Matsuda’s remark^9^) has revealed that the two major parts of pleuron, the episternum and the epimeron (Fig. 3E,F cf. Fig. 5F), originate exclusively from the subcoxa. Therefore, the subcoxal theory is embryologically substantiated.

Recent embryological studies re-evaluating the subcoxal theory with SEM have differently identified the BTA. There are two main classifications (Fig. 1): one is typified by Uchifune and Machida^11^ and another by Kobayashi *et al*.^12^.

1. In studies represented by Uchifune and Machida^11^, the BTA was misleadingly identified as the subcoxo-coxal boundary, the paratergal groove as the pleural suture, and the tergum as the subcoxa (Fig. 1). This study revealed that differentiation of the true pleural suture is accompanied by the secretion of nymphal cuticle in the final developmental phase, i.e., Stages 14–15. Following the interpretation presented by Uchifune and Machida^11^, *Tc-ser*, the most proximal domain that Coulcher *et al*.^4^ assigned to the distal margin of the subcoxa, would be expressed in the middle of the coxa (Fig. 1).
2. Kobayashi *et al*.^12^ studied the embryogenesis of the adephagan coleopteran *Carabus insulicola*, in detail with SEM. They identified the first division of the ectodermal bulge as the division between the “coxopodite” and “telopodite”; the former divides itself into the spiracle-bearing subcoxa and coxa. The tergum arises laterally (dorsally) next to the subcoxa, and the subcoxa is demarcated into the proximal, spiracle-including subcoxa 1 and the distal subcoxa 2. Kobayashi *et al*.^12^ presented a detailed description, but seem to have missed a series of rapid and drastic changes around the subcoxa that occur beneath the embryonic cuticle in the final stage of development. We notice several inexplicable points in their description. First, even in the last stages for which Kobayashi *et al*.^12^ (Figs 12 and 13) provided SEM images, the BTA remains obscure, although it should be distinct because it is a major demarcation, i.e., between the tergum and appendage. Second, in spite of being a secondary division of the subcoxa, the “paracoxal suture” identified by Kobayashi *et al*.^12^ is the most defined division. Third, based on its position relative to the spiracle, their “paracoxal suture” seems to be consistent with the BTA of *G. bimaculatus* as shown in this study. Thus, we conclude that the “paracoxal suture” identified by Kobayashi *et al*.^12^ in *C. insulicola* should be the BTA, and the structure they identified as the “subcoxa 2” is the entire subcoxa, which undergoes drastic and rapid enlargement and morphogenesis in the final developmental phase.

Although it has been not sufficiently determined, several studies suggest that spiracles may be subcoxal in origin^11,12,23,24^, and the developmental biology of *Drosophila* ^25^ suggests a close relationship between spiracles and appendages. However, this study, which succeeded in specification of the BTA in cricket embryos, showed that the spiracles arise from the antero-ventral region of the tergum, strongly suggesting their tergal origin (Figs 1,2 and 5). The prothoracic segment lacks spiracles. Instead, this study identified a small invagination with a position serially homologous to the spiracles (black arrows in Fig. 3). It may be a rudiment of the prothoracic gland as Kobayashi *et al*.^12^ suggested for *C. insulicola* embryos. A small pit identified as an invagination of the posterior tentorium was observed in the cephalic region of grown embryos at Stage 10 (“ptp” in Fig. 3A). The posterior tentorial pit is an ectodermal invagination arising where the postoccipital suture, the boundary between labial and maxillary terga, crosses the subgenal structure, which is the lateral submarginal groove of the head capsule^6,26^. Figure 3A shows that the spiracles (black arrowheads) are serially homologous with the posterior tentorial pit, which suggests the tergal origin of spiracles.

### Dual origin of wings supported

The origin of insect wings, a crucial evolutionary novelty, is one of the most attractive mysteries in insect evolution. Alternative hypotheses on the origin of wings have been long disputed, including the paranotal hypothesis, which posits that wings are tergal in origin, and the appendicular hypothesis, which posits that wings are appendicular in origin, from a limb branch such as exites and gills^27^. Recent developmental biology has proposed a third “dual origin” hypothesis mediating these alternative hypothesis^28,29^, which has been increasingly supported by recent studies^30–32^ including paleontological approach. In this concept, the bulk of the wing originates from the lateral region of the tergum paranotum and the appendage forms the hinge and musculature related to wing movement, such as flapping and folding.

**Figure 6:**
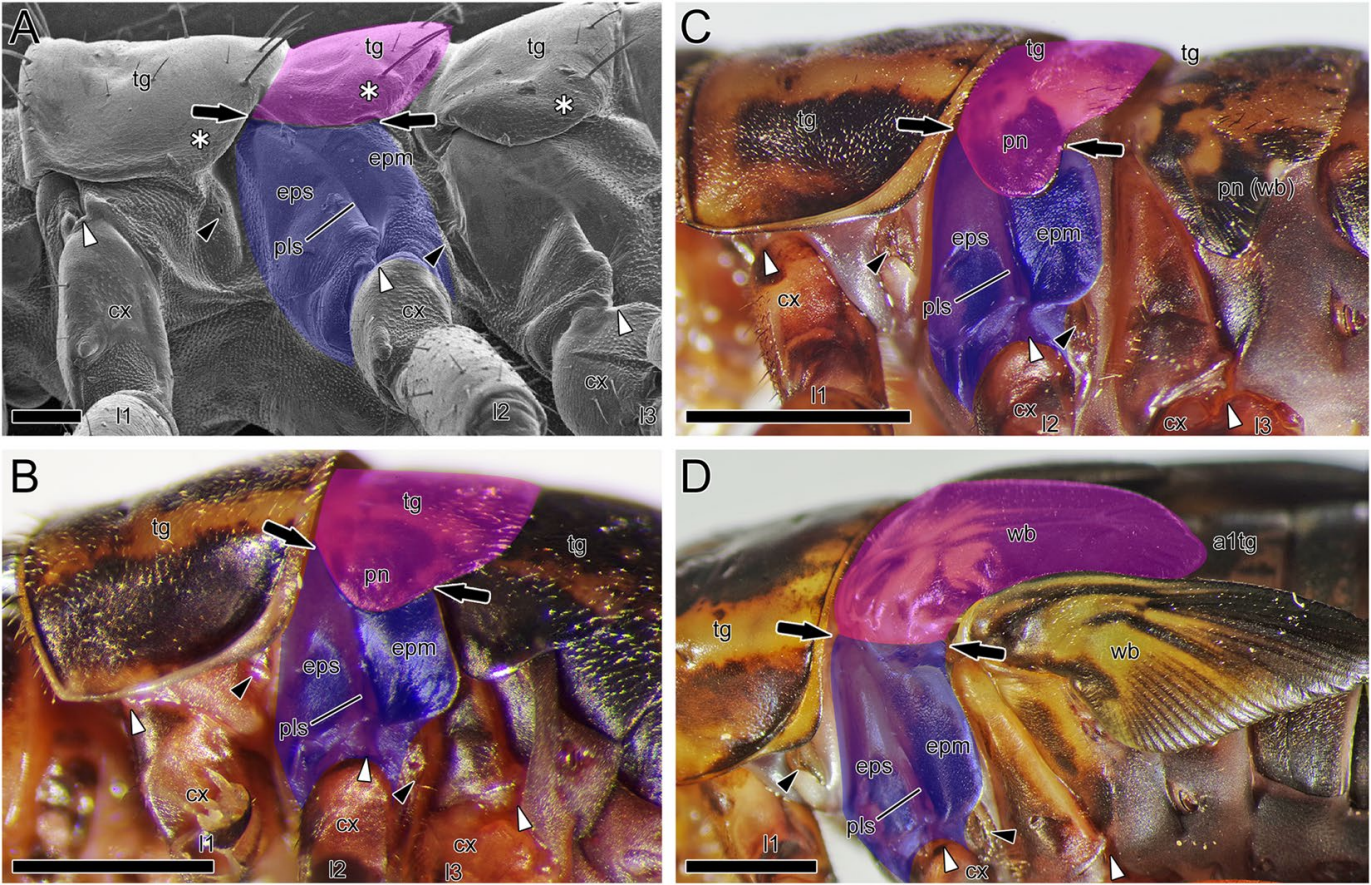
*Gryllus bimaculatus* nymphs, lateral views. (**A**) First instar nymph, SEM. (**B**) Fifth instar nymph. (**C**) Tenth instar nymph. (**D**) Eleventh instar nymph. See text. Abbreviations: a1tg, first abdominal tergum; cx, coxa; epm, epimeron; eps, episternum; l1-3, pro-, meso- and metalimbs; pls, pleural suture; pn, paranotum; tg, thoracic tergum; wb, wing bud. Color highlights: blue, pleural territory; magenta, tergal territory. Arrows, black and white arrowheads, and asterisks show the BTA, spiracles, pleuro-coxal joints, and regions corresponding to the paratergal bulge, respectively. Bars: 100 µm (**A**) ; 1 mm (**B**) ; 2 mm (**C**,**D**).

To contribute to the ongoing discussion on the origin of wings, we followed the postembryonic development of *G. bimaculatus*, which become adults after 12 moltings, i.e., adults are the 13th instar under the condition of this study (Fig. 6A–D). The tergum and the appendage differentiate side-by-side (Figs 2B and 5A), and this side-by-side positioning of the tergum and the appendage across the BTA is retained throughout development (Figs 2C–G,3A–F,5B–F and 6A–D). In the fifth instar nymph, the paratergal bulge in the lower (lateral) region of the tergum of the pterothoraxes (asterisks, e.g., in Figs 3F and 6A) starts to elongate over the pleuron, forming the paranotum or side lobe, which passes beyond the BTA (between arrows in Fig. 6B). During progressive nymphal development, this elongates further, forming the wing bud (Fig. 6C). In the 11th, i.e., the penultimate instar nymph, wing veins are clearly visible within the wing buds, especially in the metathorax. The wind buds are enlarged and turn up, so that the BTA becomes laterally exposed again (Fig. 6D). In the 12th or final instar nymph, the wing buds grow further. After the final molting, individuals become adults and the wings are complete. These findings suggest that wings are derived from extensions of paratergal bulge or the paranotum. Hence the wing proper should be tergal in origin. Wing veins form along tracheae invaded into the wing bud^33^. This study reveals that spiracles, which are openings of the tracheal system, arise in tergal territory.

This study also reveals that the pleuron should be subcoxal or appendicular in origin. The epipleurites, i.e., the basalare and subalare, are sclerites located in the epipleural region, which give insertions to the direct wing muscles, i.e., the epipleural muscles. The basalare and subalare are detached, respectively, from the episternum and epimeron of the pleuron^6^, i.e., they are the subcoxal derivatives. The epipleural muscles, i.e., basalar and subalar muscles, located behind the pleuron, which have its subcoxal origin corroborated in this study, unarguably originate in the proximal part of appendage, i.e., the coxopodite ( = subcoxa + coxa), most likely the subcoxa^6^. Therefore, we conclude that the wing proper is tergal in origin and that appendicular elements contribute to construction of the wing system. Thus, this embryological study, which was conducted to test the subcoxal theory of insect pleuron, strongly supports the dual origin hypothesis of insect wings.

Each of the alternative hypotheses on the origin of wings, i.e., the paranotal hypothesis and the appendicular hypothesis, has merits and demerits^28^. The paranotal hypothesis is consistent with the flatness and dorsal positioning of wings, but it is difficult to reason the origin of the system generating the wing movement. However, the appendicular hypothesis is consistent with the wing movement, but it is hard to explain its flatness and dorsal positioning. The third hypothesis, the dual origin hypothesis, which has been proposed based on developmental biology^28,29^ and is corroborated by this embryological study, succeeds to the merits of these alternative hypotheses, cancelling their demerits. This study explicitly explains the flatness and dorsal positioning of wings demonstrating that the wing proper is derived from the side lobes of the dorsal exoskeletal plates or paranota; it simultaneously assigns the origin of the structures responsible for working of wings to the pleural derivatives that are concluded to be appendicular in origin.

## Methods

Two-spotted crickets, *G.* *bimaculatus* were purchased from commercial suppliers, kept in a plastic container at room temperature (20–24 °C) and fed on flake fish food Tetra Fin (Spectrum Brands Japan Inc., Tokyo, Japan). Eggs laid in moistened tissue paper were collected and incubated at room temperature. Embryos were dissected in phosphate buffered saline, rinsed in fresh solution, and fixed overnight at 4 °C in Karnovsky’s fixative (2% paraformaldehyde + 2.5% glutaraldehyde 0.1 M sodium cacodylate buffer, pH 7.2). Several hundred fixed embryos were post-fixed with 1% OsO4 for 1 h. Post-fixed embryos were dehydrated in a graded ethanol series and dried with a critical point dryer Samdri®-PVT-3D (tousimis, Rockville, Maryland, USA), or dehydrated in a graded ethanol series, substituted with *t*-butanol, and dried with a freeze dryer VFD-21S (Vacuum Device, Ibaragi, Japan). Following the protocol described by Machida^16^, non-coated embryos were observed using a low-vacuum SEM SM-300 Wet-4 (TOPCON, Tokyo, Japan) at 13 Pa with an accelerating voltage of 15–30 kV. First instar nymphs were observed by SEM with the nano-suit method, according to Takaku *et al*.^17^ and Fujita *et al*.^18^. Briefly, first instar nymphs were weakly fixed with 30% ethanol to avoid shrinkage, soaked in 1% polyoxyethylene sorbitan monolaurate Tween 20 (Sigma-Aldrich, Tokyo, Japan) aqueous solution dissolved for 1 h, mounted on a specimen-stub, and observed with the same SEM under a high vacuum at an accelerating voltage of 5 kV. Hatched nymphs were reared in the same way as mentioned above, for the examination of postembryonic development. Nymphs of various instars were fixed with FAA (ethanol: formalin: acetic acid = 15: 5: 1) and photographed using a stereomicroscope MZ12 (Leica, Heerbrugg, Switzerland) equipped with a digital camera K-70 (PENTAX, Tokyo, Japan). Light micrographs taken at different focus levels were processed using the image stacking software Combine ZP^34^ to produce a single image with all sections of the specimen in focus. All image plates were prepared using Adobe Photoshop Elements (version 11.0; Adobe Systems Inc., San Jose, CA, USA) and Inkscape (version 0.92; www.inkscape.org).

## Electronic supplementary material

Supplementary figure S1
