# Supplementary material for: Embryological evidence substantiates the subcoxal theory on the origin of pleuron in insects
Source: Sci Rep. 2017 Oct 3;7:12597. doi: 10.1038/s41598-017-12728-2 (PMC5626752; doi:10.1038/s41598-017-12728-2)
Supplement: Supplementary file 1 — Supplementary figure S1 [file 41598_2017_12728_MOESM1_ESM.pdf]

Embryological evidence substantiates the subcoxal theory on the origin of pleuron in insects

Supplementary Information

Yuta Mashimo[1, 2], Ryuichiro Machida[1]

[1]Sugadaira Research Station, Mountain Science Center, University of Tsukuba, Sugadaira Kogen 1278-294, Ueda, Nagano 386-2204, Japan

machida@sugadaira.tsukuba.ac.jp (R. M.), beadsantenna@gmail.com (Y. M.)

[2]Current affiliation: Graduate School of Symbiotic Systems Science and Technology, Fukushima University, Kanayagawa 1, Fukushima, Fukushima 960-1296, Japan

Supplementary figure S1

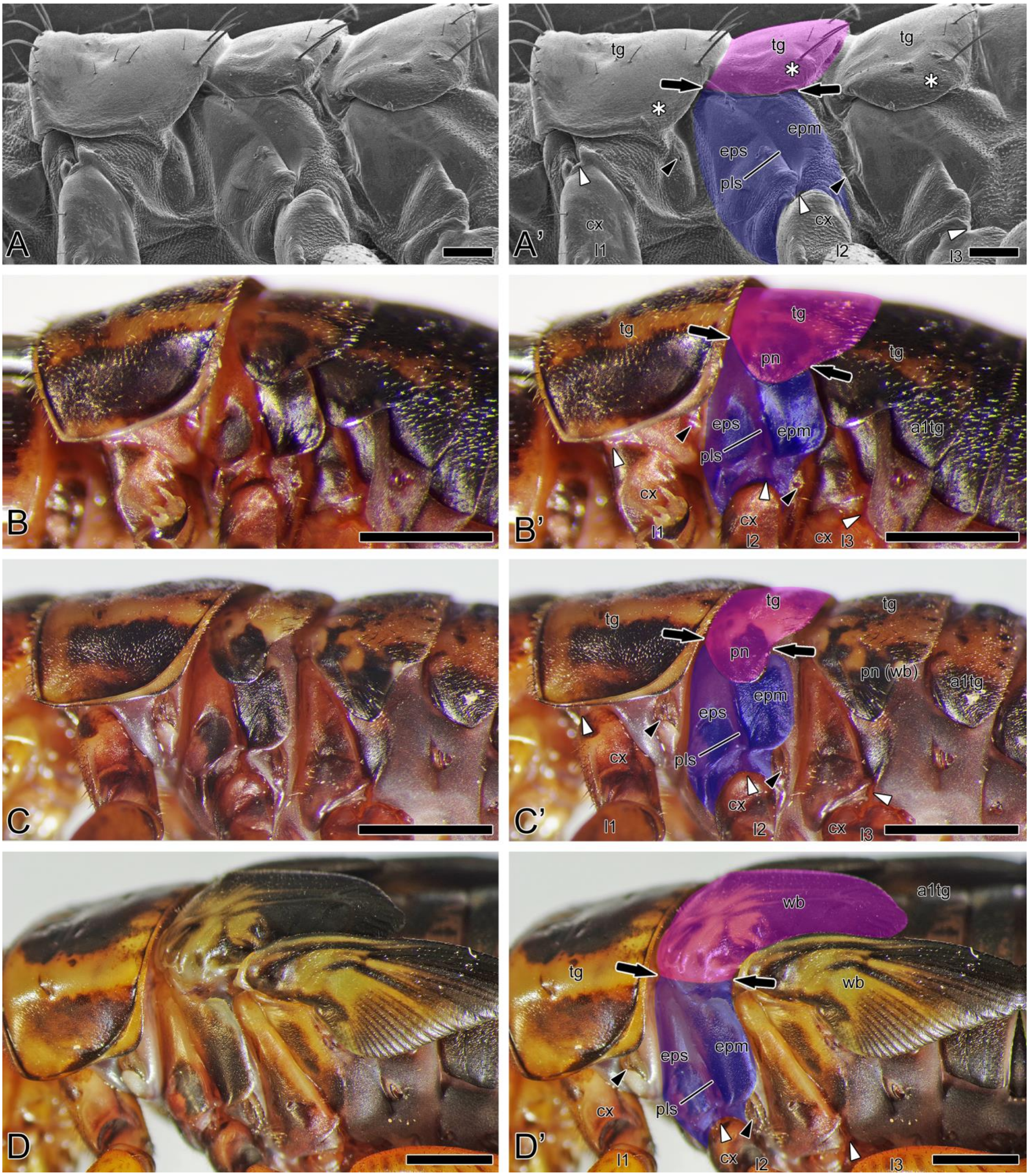

Supplementary figure S1: The same figures of *Gryllus bimaculatus* nymphs as show in Figure 6. Highlights, abbreviations, and symbols were omitted from the left panels (A–D) to show all details. (A,A’) First instar nymph, SEM. (B,B’) Fifth instar nymph. (C,C’) Tenth instar nymph. (D,D’) Eleventh instar nymph. Abbreviations: a1tg, first abdominal tergum; cx, coxa; epm, epimeron; eps, episternum; l1-3, pro-, meso- and metalimbs; pls, pleural suture; pn, paranotum; tg, thoracic tergum; wb, wing bud. Color highlights: blue, pleural territory; magenta, tergal territory. Arrows show dorsal boundary between the tergum and the appendage (see text). Black and white arrowheads and asterisks show spiracles, pleuro-coxal joints, and regions corresponding to the paratergal bulge, respectively. Bars: 100 µm (**A**); 1 mm (**B**); 2 mm (**C**,**D**).
